# Nutrient-rich versus nutrient-poor foods for depressed patients based on Iranian Traditional Medicine resources

**Published:** 2015

**Authors:** Mandana Tavakkoli-Kakhki, Saeid Eslami, Malihe Motavasselian

**Affiliations:** 1 *Department of Traditional Medicine, School of Traditional and Complementary Medicine, Mashhad University of Medical Sciences, Mashhad, Iran*; 2 *Pharmaceutical Research Center, School of Pharmacy, Mashhad University of Medical Sciences, Mashhad, Iran*; 3 *Department of Medical Informatics, Academic Medical Center, University of Amsterdam, Amsterdam, the Netherlands*

**Keywords:** *Medicine*, *Traditional*, *Depression*, *Food*

## Abstract

**Objectives:**

Considering the positive effects of certain nutrients on depression, increasingly prevalent in the contemporary societies, we investigated the nutritional content of prescribed and prohibited foodstuffs for depressed patients in Iranian Traditional Medicine resources.

**Materials and Methods:**

In order to conduct the study, credible sources of Iranian Traditional Medicine were primarily reviewed for the prescribed and prohibited foodstuffs for depressed patients. USDA database, as a well-known and valuable source, was then visited to determine the amount of effective nutrients in each foodstuff. Finally, the obtained amounts were compared with each other in three food groups, namely vegetables, fruits and nuts and also high protein products.

**Results:**

In Iranian Traditional Medicine texts, the following are prescribed for depression management: basil, coriander, spinach, lettuce, squash, peppermint, dill, chicory, celery, chard, quince, cucumber, watermelon, grape, peach, pomegranate, banana, apple, currant, pistachio, dried fig, almond, egg, chicken, lamb, and trout; cabbage, eggplant, onion, garlic, broad beans, lentils, and beef, meanwhile, are prohibited. In this regard, the effective nutritional content of these foodstuffs was obtained and then compared in the three food groups.

**Conclusion:**

This study revealed that spinach, lettuce, chicory, and squash (vegetables), pomegranate and almond (fruits and nuts) and ultimately trout (high protein products) are the best effective foodstuffs on depressed patients from nutritional content aspect.

## Introduction

Major depressive disorder (unipolar major depression), dysthymic disorder, and minor depressive disorder represent depressive syndromes which are distinguished by their type and number of symptoms as well as their duration. 

Depressive symptoms may include depressed mood states, loss of interest or pleasure in most or all activities, insomnia or hypersomnia, changes in appetite or weight, psychomotor retardation or agitation, low energy, poor concentration, thoughts of worthlessness or guilt, and recurrent thoughts about death or suicide (American Psychiatric Association, 2000 ▶). Accordingly, depression is a major public health issue associated with increased functional disability (Donohue and Pincus, 2007 ▶). Furthermore, depressive disorders (Richards, 2011 ▶) and some of their significant complications such as cardiovascular diseases (Nemeroff and Goldschmidt-Clermont, 2012 ▶), Alzheimer (Wint, 2011 ▶), and diabetes (Silva et al., 2012 ▶) are becoming more prevalent in modern societies. Nowadays, pharmacotherapy and psychotherapy are the more widespread treatments for depressive disorders. In the scope of pharmacotherapy, long-term drug therapy leads to impairments in the intellectual and cognitive functions alongside other considerable side effects. To this end, public tendency for alternative or complementary treatments with fewer side effects has been recently intensified. (Sadock et al., 2007 ▶).

Iranian Traditional Medicine (ITM) as a Complementary and Alternative Medicine (CAM) involves several non-pharmacological methods of treating melancholia (a psychotic type of depression) like food therapy, aromatherapy, color therapy, phototherapy, voice therapy, exercise, massage, sleep strategies, bathroom strategies, anointment, sexual intercourse, puking, feces laxation, induced menstruation, game therapy, narrative therapy, and traveling. One of the most notable of these methods is food therapy which includes singular and combined foodstuffs, and nutrition rules (AhwaziArjani, 1973 ▶; Ibn-e Sina, 2005 ▶; JorJani, 1976 ▶). 

Recent studies have revealed the preventive effects of such diets as Mediterranean diet on depression (Sánchez-Villegas et al., 2006 ▶) or its complications (Antonogeorgos et al., 2012 ▶). This type of diet includes vegetables, fruits, grains, frijol, nuts, dairy, olive oil, and aquatic with no priority for any foodstuff (Sánchez-Villegas et al., 2006 ▶). ITM resources, on the other hand, provide melancholic patients with specific kinds of vegetables (basil, coriander, spinach, lettuce, squash, peppermint, dill, chicory, celery, chard), fruits (quince, cucumber, watermelon, grape, peach, pomegranate, banana, apple), dry fruits and nuts (currant, pistachio, dried fig, almond), aquatic (trout), and frijoles, with the exception of broad beans and lentils(Tavakkoli et al., 2014a ▶).

On the other hand, the positive effects of some nutrients have already been investigated on depression: certain amino acids (Bressa, 1994 ▶; Firk and Markus, 2009 ▶; Roiser et al., 2005 ▶), fat soluble vitamins (Owen et al., 2005 ▶; Parker and Brotchie, 2011 ▶), water soluble vitamins (Almeida et al., 2010 ▶; Benton et al., 1995 ▶; Davison and Kaplan, 2012 ▶; Gilbody et al., 2007 ▶; Murakami et al., 2010 ▶; Williams et al., 2005 ▶; Zhang et al., 2011 ▶), minerals (Davison and Kaplan, 2012 ▶; Eby and Eby, 2009 ▶; Lai et al., 2012 ▶; McClung et al., 2009 ▶; Torres et al., 2008 ▶), carbohydrates (Achten et al., 2004 ▶), and fiber (Logan, 2006 ▶). Therefore, the main objective of the present study was to investigate the nutritional content of ITM’s prescribed or prohibited foodstuffs for melancholia as a type of depression.

## Materials and Methods

In this study, three authoritative ITM references, namely Kamel al-Sanaat al-Tibbyyah, Al-Qanun fi al-Tibb, and Zakhireh Kharazmshahi were carefully reviewed to extract prohibited and prescribed foodstuffs concerning depression management. Based on these resources, melancholia can be considered as a psychotic type of depression. Moreover, according to the food-based strategies of controlling melancholic depression, the following are considered as helpful: basil *(Ocimum basilicum),* coriander *(Coriandrum sativum),* spinach *(Spinacia oleracea),* lettuce *(Lactuca sativa),* squash *(Cucurbita moschata),* peppermint *(Mentha piperita),* dill *(Anethum graveolens),* chicory *(Cichorium intybus),* celery *(Apium graveolens),* chard *(Beta vulgaris),* quince *(Cydonia oblonga),* cucumber *(Cucumis sativus),* watermelon *(Citrullus lanatus),* grape *(Vitis vinifera),* peach *(Prunus persica),* pomegranate *(Punica granatum),* banana *(Musa acuminata),* apple *(Malus domestica),* currant *(Vitis vinifera),* pistachio *(Pistacia vera),* dried fig *(Ficus carica),* almond *(Prunus dulcis),* egg, chicken, lamb, and trout; however, cabbage *(Brassica oleracea),* eggplant *(Solanumme longena),* onion *(Allium cepa),* garlic *(Allium sativum), *broad beans *(Vicia faba),* lentils *(lens culinaris),* and beef are deemed as harmful, thus prohibited (Ahwazi Arjani, 1973 ▶; Ibn-e Sina, 2005 ▶; JorJani, 1976 ▶). It should be noted that the scientific names of the vegetarian foodstuffs were retrieved from USDA database (United States Department of Agriculture, 2011).

Next, we determined the amount of the following nutrients (Which are effective on depressed patients) in 100g of each foodstuff employing the already-mentioned database: phenylalanine (Roiser et al., 2005 ▶), methionine (Bressa, 1994 ▶), tryptophan (Firk and Markus, 2009 ▶), vitamin D (Parker and Brotchie, 2011 ▶), vitamin E (Owen et al., 2005 ▶), vitamin B12 (Almeida et al., 2010 ▶; Davison and Kaplan, 2012 ▶; Murakami et al., 2010 ▶), folate (Almeida et al., 2010 ▶; Davison and Kaplan, 2012 ▶; Gilbody et al., 2007 ▶; Murakami et al., 2010 ▶), vitamin B6 (Almeida et al., 2010 ▶; Davison and Kaplan, 2012 ▶; Murakami et al., 2010 ▶; Williams et al., 2005 ▶), niacin (Davison and Kaplan, 2012 ▶), riboflavin (Benton et al., 1995 ▶; Davison and Kaplan, 2012 ▶; Murakami et al., 2010 ▶), thiamin (Benton et al., 1995 ▶), vitamin C (Zhang et al., 2011 ▶), zinc (Davison and Kaplan, 2012 ▶; Lai et al., 2012 ▶), potassium (Davison and Kaplan, 2012 ▶; Torres et al., 2008 ▶), magnesium (Davison and Kaplan, 2012 ▶; Eby and Eby, 2009 ▶), iron (Davison and Kaplan, 2012 ▶; McClung et al., 2009 ▶), calcium (Davison and Kaplan, 2012 ▶; Torres et al., 2008 ▶), carbohydrates (Achten et al., 2004 ▶), and fiber (Logan, 2006 ▶).

It is worth mentioning that, considering the positive effect of polyunsaturated fatty acids on depression (Kraguljac, 2009 ▶), the omega-3 content of the target foodstuffs has also been investigated in another study (Tavakkoli et al., 2014 ▶). However due to the importance of this topic, we referred to the findings of mentioned study; wherever it is necessary in the present research.

If a specific nutrient amount of a foodstuff was not specified in USDA database, we would try to approximate the intended amount based on the information given for an alternative foodstuff in USDA database. The needed data, however, were extracted from a supplementary reference (Dorosti and Tabatabai, 2007 ▶) in the case where USDA method was not feasible. It was impossible to obtain the amount of coriander, pomegranate, quince amino acids and quince vitamin E through these two methods, hence their exclusion from calculation and analysis. 

In the second step, each nutrient’s amount in 100 g of each foodstuff was divided into the necessary daily value of each nutrient determined based on the specified values ​​for an adult male (Mahan and Escott-Stump, 2008 ▶). After that, the result was multiplied by 100 to determine the percentage of each nutrient’s necessary daily value provided by 100 g of each foodstuff. In this way, the percentage of “provided nutrient per 100 g” was calculated for every nutrient in each foodstuff.

In the third step, the percentage of “provided nutrient per 100 g” of each foodstuff for every nutrient was divided into 100. The results were then multiplied by specified serving sizes (g) for each foodstuff (Clínica Mayo, 2002 ▶; Ghaffarpoor et al., 2001 ▶) so as to determine the percentage of necessary daily value of each nutrient provided by a serving size of each foodstuff. In this way, the percentage of “Provided Nutrient per Serving size” (PNS) was obtained for every nutrient contained in each foodstuff. 

Next, PNS values were separately calculated in five groups of amino acids, fat soluble vitamins, water soluble vitamins, minerals and carbohydrates. for each foodstuff. 

**Statistical analysis**

In the vegetables group, the PNS values of​​ amino acids, fat soluble vitamins, water soluble vitamins, minerals, and carbohydrates were compared in both prescribed and prohibited foodstuffs using t-test (SigmaPlot12), where P-value <0.05 was considered as statistically significant. 

## Results

Since it was necessary to separately compare the nutritional content of the prescribed and prohibited foodstuffs, the results were divided to three groups of 1) vegetables, 2) fruits, dry fruits and nuts, and 3) high protein products.

**Vegetables**

Tables 1 and 2 indicate the PNS values of the​​ five groups of amino acids, fat soluble vitamins, water soluble vitamins, minerals, and carbohydrates along with total nutrients contained in both prescribed and prohibited vegetables.

In addition, table 3 shows the results of the comparisons made among the PNS averages of amino acids, fat soluble vitamins, water soluble vitamins, minerals, carbohydrates, and total nutrients between prescribed and prohibited vegetables. According to the obtained results, the prescribed vegetables group surpassed the prohibited vegetables group in minerals and fat-soluble vitamins tracks (Table 3). 

Additionally, investigations without using software in the cases of statistically insignificant were also carried out as follows:

In the case of amino acids, the difference between the two groups was in favor of the prescribed group, but it was not significant. On the other hand, PNS values of amino acids in spinach, chicory, and lettuce were more than eggplant, onion, garlic, and cabbage (Table 1 and 2).

In the case of water soluble vitamins, the difference between the two groups was in favor of the prescribed group, but it was not significant. On the other hand, PNS values of water soluble vitamins in spinach, chicory, lettuce, squash, and dill were more than eggplant, onion, garlic and cabbage (Table 1 and 2).

In the case of carbohydrates, the difference between the two groups was in favor of the prohibited group but it was not significant. On the other hand, PNS values of carbohydrates in chicory and squash were more than eggplant, onion, garlic, and cabbage (Table 1 and 2).

Regarding omega-3 content, the ratio of omega-6 to omega-3 in mint, spinach, basil, lettuce, and squash was less than all the prohibited vegetables. Nevertheless, coriander, dill, chicory, celery and chard, which are actually prescribed in this group, do not have the same properties. It is worth mentioning that the fewer the amount of this ratio in a foodstuff the more desirable it will be; and the best defined ratio of omega-6 to omega-3 in one foodstuff was 2:1 or 3:1 (Mahan and Escott-Stump, 2008 ▶).

The results showed that spinach and lettuce, with higher PNS values of amino acids and water soluble vitamins and also lower ratio of omega-6 to omega-3, surpassed all the prohibited vegetables in these three tracks. Furthermore, chicory contained higher PNS values of amino acids, water soluble vitamins, and total carbohydrates, hence surpassing all of the prohibited vegetables in these three tracks.

**Table 1:** Provided nutrient per serving size (PNS) based on the percentage for each nutrient in prohibited vegetables

**Cabbage**	**Garlic**	**Onions**	**Eggplant**	**Ingredients**
2.43	1.46	1.72	5.69	**Phenylalanine**
0.98	0.65	0.15	1.57	**Methionine**
3.90	2.46	4.50	5.55	**Tryptophan**
7.31	4.58	6.37	12.81	**Amino acids**
0.00	0.00	0.00	0.00	**Vitamin D**
0.74	0.04	0.09	2.59	**Vitamin E**
0.74	0.04	0.09	2.59	**Fat soluble vitamins**
0.00	0.00	0.00	0.00	**Vitamin B-12**
8.00	0.06	3.21	7.13	**Folate**
7.10	7.44	6.23	8.37	**Vitamin B-6**
1.09	0.34	0.49	5.26	**Niacin**
2.29	0.66	14.02	3.69	**Riboflavin**
3.78	1.31	2.59	4.21	**Thiamin**
30.26	2.71	5.55	3.17	**Vitamin C**
52.51	12.52	32.08	31.83	**Water soluble vitamins**
1.22	0.83	1.04	1.89	**Zinc**
2.69	0.67	2.10	6.31	**Potassium**
2.13	0.47	1.61	4.32	**Magnesium**
4.37	1.66	1.77	3.73	**Iron**
2.98	1.42	1.55	0.91	**Calcium**
13.38	5.04	8.07	17.15	**Minerals**
3.32	1.99	4.85	5.86	**Carbohydrates**
77.26	24.17	51.46	70.24	**Total**

**Table 2:** Provided nutrient per serving size (PNS) based on the percentage for each nutrient in prescribed vegetables

coriander	Dill	Basil	Peppermint	Celery	Squash	Lettuce	Chicory	Chard	Spinach	Ingredients
-	3.32	0.97	0.94	1.63	3.98	8.42	7.53	6.29	7.37	**Phenylalanine**
-	0.60	0.29	0.28	0.44	1.32	2.64	1.98	1.17	3.26	**Methionine**
0.00	7.25	2.61	2.54	5.50	11.96	17.48	36.08	11.99	21.03	**Amino acids**
0.00	0.00	0.00	0.00	0.00	0.00	0.00	0.00	0.00	0.00	**Vitamin D**
8.33	5.67	0.39	1.60	1.44	9.60	2.20	27.12	7.06	7.58	**Vitamin E**
8.33	5.67	0.39	1.60	1.44	9.60	2.20	27.12	7.06	7.58	**Fat soluble vitamins**
0.00	0.00	0.00	0.00	0.00	0.00	0.00	0.00	0.00	0.00	**Vitamin B-12**
7.75	18.75	1.24	1.37	7.20	6.75	14.25	49.50	1.96	27.16	**Folate**
5.73	7.12	0.87	0.48	4.55	11.85	10.38	14.54	4.26	8.40	**Vitamin B-6**
3.48	4.91	0.41	0.51	1.60	7.50	3.52	5.63	1.40	2.53	**Niacin**
6.23	11.38	0.43	0.98	3.51	1.54	9.23	13.85	3.88	8.14	**Riboflavin**
2.79	2.42	0.21	0.33	1.40	8.33	8.75	9.00	1.87	3.64	**Thiamin**
15.00	47.22	1.46	1.70	2.76	23.33	15.33	48.00	18.67	17.48	**Vitamin C**
40.98	91.80	4.62	5.36	21.02	59.30	61.46	140.51	32.03	67.36	**Water soluble vitamins**
2.27	4.14	0.54	0.48	0.95	1.36	2.45	6.87	1.83	2.70	**Zinc**
5.54	7.85	0.09	0.58	4.43	7.49	6.19	16.09	4.52	6.65	**Potassium**
3.10	6.55	1.11	0.91	2.10	8.10	4.64	12.86	10.80	10.53	**Magnesium**
11.06	41.19	2.89	3.05	2.00	8.75	16.13	20.25	12.60	18.97	**Iron**
3.35	10.40	1.29	1.17	3.20	4.80	5.40	18.00	2.86	5.54	**Calcium**
25.32	70.12	5.92	6.19	12.67	30.50	34.81	74.06	32.60	44.39	**Minerals**
1.41	2.70	0.15	0.55	1.83	8.99	3.31	6.51	1.61	1.56	**Carbohydrates**
76.05	177.54	13.69	16.25	42.45	120.36	119.27	284.28	85.29	141.93	**Total**

**Table 3:** Comparison between PNS averages in prohibited and prescribed vegetables using t-test

Ingredients	PNS averages in prohibited vegetables (%) (N=4)	PNS averages in prescribed vegetables (%) (N-10)	P-value
Amino acids	7	15	0.08
Fat soluble vitamins	0.8	8.6	0.04
Water soluble vitamins	32	64	0.07
Minerals	10	40	0.01
Carbohydrates	4	3.4	0.37
Total	55	130	0.04

Squash with higher PNS amounts of water soluble vitamins and total carbohydrates and also lower omega-6 to omega-3 ratio, surpassing all the prohibited vegetables in these three tracks. 

Dill, with higher PNS values of water soluble vitamins exceeded all of the prohibited vegetables in this particular track. Also, peppermint and basil with lower omega-6 to omega-3 ratio surpassed all of the prohibited vegetables in this track. Consequently, spinach, lettuce, chicory, and squash were more effective than all of the prescribed vegetables through three other tracks except for minerals and fat soluble vitamins (spinach and lettuce: amino acids, water soluble vitamins, omega-6 to omega-3 ratio chicory: amino acids, water soluble vitamins, total carbohydrates Squash: water soluble vitamins, total carbohydrates, omega-6 to omega-3 ratio).

**Fruits, dry fruits, and nuts**

Among the fruits, the total PNS decreases in pomegranate, peach, grape, banana, watermelon, quince, cucumber, and apple respectively. But it should be considered that the amount of amino acids in pomegranate and quince and also the amount of vitamin E in quince were not specified. Among the dried fruits and nuts, the total PNS decreased in almond, pistachio, currant, and dried fig respectively (Table 4 and 5). Nevertheless, in this section, it was assumed that the more the total PNS value of one foodstuff is, the more effective that foodstuff would be on depressed patients.

**Table 4:** Provided nutrient per serving size (PNS) based on the percentage for each nutrient in prescribed fruits

Quince	Watermelon	Peach	Banana	Apple	Cucumber	Grape	Pomegranate	Ingredients
-	2.76	3.44	3.84	0.83	4.62	2.79	-	**Phenylalanine**
-	1.19	1.95	0.68	0.15	1.93	1.42	-	**Methionine**
-	6.00	8.46	3.29	0.64	4.87	7.54	-	**Tryptophan**
-	9.94	13.85	7.81	1.62	11.41	11.76	-	**Amino acids**
0.00	0.00	0.00	0.00	0.00	0.00	0.00	0.00	**Vitamin D**
-	0.60	8.64	0.51	1.62	0.29	1.82	7.50	**Vitamin E**
-	0.60	8.64	0.51	1.62	0.29	1.82	7.50	**Fat soluble vitamins**
0.00	0.00	0.00	0.00	0.00	0.00	0.00	0.00	**Vitamin B-12**
0.86	1.35	1.78	3.84	1.01	5.11	0.72	17.82	**Folate**
3.53	6.23	3.42	21.68	4.26	5.73	9.53	10.82	**Vitamin B-6**
1.43	2.00	8.95	3.19	0.77	3.38	1.69	3.44	**Niacin**
2.65	2.91	4.24	4.31	2.70	2.81	7.75	7.65	**Riboflavin**
1.91	4.95	3.55	1.98	1.91	3.77	8.28	10.47	**Thiamin**
19.13	16.20	13.02	7.42	6.90	5.19	5.12	21.26	**Vitamin C**
29.51	33.64	34.95	42.43	17.55	25.98	33.09	71.46	**Water soluble vitamins**
0.42	1.64	2.74	1.05	0.49	2.26	0.92	5.97	**Zinc**
4.81	4.29	7.18	5.85	3.07	4.22	5.85	9.42	**Potassium**
2.19	4.29	3.81	4.94	1.61	4.17	2.40	5.36	**Magnesium**
10.04	5.40	5.55	2.50	0.20	4.02	6.48	7.04	**Iron**
1.26	1.26	1.07	0.38	0.81	2.04	1.44	1.88	**Calcium**
18.72	16.87	20.35	14.71	6.18	16.71	17.09	29.66	**Minerals**
13.51	10.45	13.03	13.49	14.34	2.43	20.05	26.99	**Carbohydrates**
61.73	71.51	90.82	78.96	41.31	56.82	83.81	135.61	**Total**

**Table 5:** Provided nutrient per serving size (PNS) based on the percentage for each nutrient in prescribed dried fruits

Currant	Pistachio	Almond	Dried fig	Ingredients
0.34	15.06	22.86	0.74	**Phenylalanine**
0.60	5.15	3.32	0.36	**Methionine**
0.37	18.07	20.38	0.91	**Tryptophan**
1.31	38.28	46.56	2.02	**Amino acids**
0.00	0.00	0.00	0.00	**Vitamin D**
0.07	2.15	34.96	0.22	**Vitamin E**
0.07	2.15	34.96	0.22	**Fat soluble vitamins**
0.00	0.00	0.00	0.00	**Vitamin B-12**
0.24	1.79	2.50	0.22	**Folate**
2.19	18.31	2.20	0.78	**Vitamin B-6**
0.97	1.14	4.23	0.37	**Niacin**
1.05	1.72	15.60	0.61	**Riboflavin**
1.28	10.15	3.52	0.68	**Thiamin**
0.50	0.87	0.00	0.13	**Vitamin C**
6.22	33.97	28.05	2.78	**Water soluble vitamins**
0.58	2.80	5.60	0.48	**Zinc**
1.82	3.05	3.00	1.39	**Potassium**
0.94	4.03	12.76	1.55	**Magnesium**
3.91	6.86	9.30	2.44	**Iron**
0.83	1.47	5.28	1.56	**Calcium**
8.07	18.22	35.94	7.41	**Minerals**
5.47	2.96	3.33	4.72	**Carbohydrates**
21.15	95.58	148.84	17.16	**Total**

It should be noted that according to the study of the prescribed fruits, dry fruits and nuts considering their omega-3 content, among the prescribed fruits, the ratio of omega-6 to omega-3 in cucumber is less than other prescribed fruits. Also, among the prescribed dried fruits and nuts the ratio of omega-6 to omega-3 in pistachio is less than other prescribed dried fruits and nuts.

**High protein products**

In this group, PNS values of fat soluble vitamins in chicken and egg were more than in broad beans, lentils, and beef. Moreover, lamb had higher PNS amounts of water soluble vitamins than broad beans, lentils, and beef. Also, PNS values of amino acids, fat soluble vitamins, and water soluble vitamins were more in trout than in broad beans, lentils, and beef (Table 6).

Regarding omega-3 content, lamb had a lower omega-6 to omega-3 ratio than broad beans and lentils, yet a higher ratio than beef. Egg, chicken, and trout, also prescribed in this group, did not have the same properties.

The results showed that trout by its amino acids, fat soluble and water soluble vitamins content, lamb, with its water soluble vitamins content and finally chicken and egg with their fat soluble vitamins content, were more effective on depression than broad beans, lentils and beef.

## Discussion

The main finding of this study is as follows: in the vegetables group, spinach, lettuce, chicory and squash, in the fruits and nuts group, pomegranate and almond and ultimately in the high protein products group, trout were the best effective foodstuffs on depressed patients considering their nutrients content. But it should be noted that cucumber and pistachio would be more effective than other fruits and nuts if only the omega-6 to omega-3 ratio was to be considered.

This research also revealed that not all the ITM-prescribed foodstuffs for depressed patients are nutrient-rich and not all the ITM-prohibited foodstuffs are nutrient-poor, suggesting that other active ingredients are to be analyzed in the aforementioned foodstuffs.

**Table 6:** Provided nutrient per serving size (PNS) based on the percentage for each nutrient in high protein products

Prescribed				Prohibited			Ingredients
Trout	Lamb	Chicken	Egg	Beef	Lentils	Broad beans	
71.13	60.64	59.63	36.64	62.47	67.90	65.92	**Phenylalanine**
58.09	41.16	43.97	22.05	44.07	12.64	13.71	**Methionine**
95.30	81.03	79.97	41.99	83.53	57.75	68.89	**Tryptophan**
224.52	182.83	183.56	100.67	190.07	138.28	148.52	**Amino acids**
271.99	1.71	1.71	21.12	1.70	0.00	0.00	**Vitamin D**
13.34	1.14	1.88	3.70	1.82	1.71	0.20	**Vitamin E**
285.33	2.85	3.60	24.82	3.52	1.71	0.20	**Fat soluble vitamins**
153.24	91.57	11.42	19.58	79.83	0.00	0.00	**Vitamin B-12**
2.35	4.06	1.29	6.20	1.28	62.59	61.94	**Folate**
22.37	9.21	21.75	6.90	24.89	21.71	16.49	**Vitamin B-6**
29.76	30.04	33.53	0.25	18.52	8.51	10.37	**Niacin**
5.92	13.81	11.01	18.56	9.83	8.48	15.00	**Riboflavin**
8.55	7.84	8.14	1.76	6.39	38.03	27.09	**Thiamin**
2.76	0.00	0.00	0.00	0.00	2.56	0.91	**Vitamin C**
224.95	156.52	87.14	53.26	140.73	141.88	131.80	**Water soluble vitamins**

There are several limitations in this study that may affect the generalizability of the findings. One of the limitations is that only USDA database was used in determining the nutritional amounts. USDA database, however, is one of the most valuable and well-known databases in this domain; therefore, the odds that the obtained results were affected by this factor are low. The other point concerning factors affecting generalization is that the assumption in the research was that all nutrients would have a same effect on depression while, in fact, this is not exactly the case. 

In addition, previous studies investigated the interaction among certain nutrients (Novitskala, 1950 ▶; Reeves and DeMars, 2004 ▶) that were not actually considered in the present study. Moreover, considering the strong effect of insoluble fibers on depression, it was necessary to study the values of this case in the prohibited and prescribed vegetables, fruits, and nuts; the USDA database, however, only contained the values of total fiber. The mentioned points would be the unavoidable limitations of this research work.

Many studies have been conducted regarding the nutrition of patients suffering from depression. These studies can be classified in two groups: the first group suggests helpful nutrients while the second proposes appropriate diets or foods.

In the first group, there are some studies that illustrate the positive effects of the following nutrients on depression: phenylalanine (Roiser et al., 2005 ▶), methionine (Bressa, 1994 ▶), tryptophan (Firk and Markus, 2009 ▶), vitamin D (Parker and Brotchie, 2011 ▶), vitamin E (Owen et al., 2005 ▶), vitamin B12 (Almeida et al., 2010 ▶; Davison and Kaplan, 2012 ▶; Murakami et al., 2010 ▶), folate (Almeida et al., 2010 ▶; Davison and Kaplan, 2012 ▶; Gilbody et al., 2007 ▶; Murakami et al., 2010 ▶), vitamin B6 (Almeida et al., 2010 ▶; Davison and Kaplan, 2012 ▶; Murakami et al., 2010 ▶; Williams et al., 2005 ▶), niacin (Davison and Kaplan, 2012 ▶), riboflavin (Benton et al., 1995 ▶; Davison and Kaplan, 2012 ▶; Murakami et al., 2010 ▶), thiamin (Benton et al., 1995 ▶), vitamin C (Zhang et al., 2011 ▶), zinc (Davison and Kaplan, 2012 ▶; Lai et al., 2012 ▶), potassium (Davison and Kaplan, 2012 ▶; Torres et al., 2008 ▶), magnesium (Davison and Kaplan, 2012 ▶; Eby and Eby, 2009 ▶), iron (Davison and Kaplan, 2012 ▶; McClung et al., 2009 ▶), calcium (Davison and Kaplan, 2012 ▶; Torres et al., 2008 ▶), carbohydrates (Achten et al., 2004 ▶), and fiber (Logan, 2006 ▶). In these studies, the focus is for the most part on usual or enriched diets and their effects, specified with regard to their nutritional content. 

For example when the amount of a desirable nutrient in a diet is low and the incidence of depression is high, the implication is that the paucity of that nutrient leads to the increasing of depression. On the other hand, when the amount of a desired nutrient in a diet is adequate and the incidence of depression is low, it is concluded that the adequate supplying of that nutrient leads to decreasing of depression. It is noticeable that, the aim of these studies is to determine the helpful nutrients, not the particular foodstuffs providing them helpful nutrients.

The second group of the studies introduces appropriate diets or foodstuffs for these patients. In this group the effect of a specific diet or foodstuff has been investigated on depressed patients. For instance, it has been shown that Mediterranean diet (including vegetables, fruits, grains, frijoles, nuts, dairy, olive oil, and aquatic) has certain positive effects on depression (Sánchez-Villegas, 2006 ▶). What seems to be lacking in such studies, however, is the investigation of the effective compounds and their nutritional content in the prescribed foodstuffs.

Unlike the aforementioned groups, the present research introduced prescribed vegetables, fruits, dry fruits, nuts, and high protein products alongside investigating their nutritional content. Prohibited vegetables and frijoles were also proposed along with their nutritive content. In the second group of studies, however, all of the vegetables and frijoles were prescribed without mentioning any exceptions in the framework of the Mediterranean diet (Sánchez-Villegas, 2006 ▶). These can be considered as the strong points of the present study.

Certain studies were done in the second group regarding the effectiveness of fish as a providing source of omega-3 fatty acids (Bountziouka, 2009 ▶). According to the obtained results, the content of amino acids, fat soluble vitamins, and water soluble vitamins in trout is more than beef (a prohibited foodstuff). Nonetheless, the omega-6 to omega-3 ratio in beef (2:1) is higher than it is in trout (8:1). Since the approximate ratio of omega-6 to omega-3 in wild trout is 2:1, this contradiction could be related to the selection of the framed trout instead of the wild trout in the calculations (Tavakkoli et al., 2014b ▶).

The present study offers spinach, lettuce, chicory, squash, pomegranate, almond and wild trout as foodstuffs having the highest effect on patients suffering from depression, a fact that can be considered by clinicians.

Since in this research work only the effective nutrients on depressed patients have been investigated; study on the possible impacts of other effective compounds in the intended foodstuffs would lead to more accurate results. Moreover, it would be remarkably useful to design observational or interventional studies based on the main findings of the present research.
